# Plant Alkaloids as an Emerging Therapeutic Alternative for the Treatment of Depression

**DOI:** 10.3389/fphar.2016.00028

**Published:** 2016-02-15

**Authors:** Sadia Perviz, Haroon Khan, Aini Pervaiz

**Affiliations:** Department of Pharmacy, Abdul Wali Khan University MardanMardan, Pakistan

**Keywords:** plants, alkaloids, antidepressant effects, multiple mechanisms, future therapies

## Abstract

Depression is a heterogeneous mood disorder that has been classified and treated in a variety of ways. Although, a number of synthetic drugs are being used as standard treatment for clinically depressed patients, but they have adverse effects that can compromise the therapeutic treatments and patient's compliance. Unlike, synthetic medications, herbal medicines are widely used across the globe due to their wide applicability and therapeutic efficacy associated with least side effects, which in turn has initiated the scientific research regarding the antidepressant activity. This review is mostly based on the literature of the last decade, aimed at exploring the preclinical profile of plant-based alkaloids (the abundant secondary metabolite) as an emerging therapy for depression.

## Introduction

Depression is a state of low mood and aversion to activity that can affect a person's thoughts, behavior, feelings and sense of well-being (Sandra, 1997; Rosenbaum et al., 2016). People with depressed mood can feel sad, anxious, empty, hopeless, helpless, worthless, guilty, irritable, ashamed, or restless. They may lose interest in activities that were once pleasurable, experience loss of appetite or overeating (Ingram, 2016). Physical changes also occur, particularly in severe, vital, or melancholic depression. These include insomnia or hypersomnia; altered eating patterns, with anorexia and weight loss or sometimes overeating; decreased energy and libido; and disruption of the normal circadian and ultradian rhythms of activity, body temperature, and many endocrine functions (Tondo et al., 2003). The depressed mood is a feature of some psychiatric syndromes such as major depressive disorder, but it may also be a normal reaction to living events such as bereavement, a symptom of some bodily ailments or a side effect of some drugs and medical treatments. Patients with major depression have symptoms that reflect changes in the brain, monoamine neurotransmitters, specifically norepinephrine, serotonin, and dopamine (Gold et al., 1988). Some features of depressive disorder overlap those of the anxiety disorders, including severe phobias, generalized anxiety disorder, social anxiety disorder, post-traumatic stress disorder, and obsessive-compulsive disorder (Muhammad et al., 2013). The disorder is also often associated with suicide and there are between 10 and 20 million suicide attempt every year (Calvó-Perxas et al., 2016; Gadassi and Mor, 2016). According to World Health Report, about 450 million people suffer from a mental or behavioral disorder. This amounts to 12.3% of the global burden of disease and predicted to rise up to 15% by 2020 (Ridout et al., 2016).

The current antidepressant drugs are facing many challenges as mentioned by Alexander and Preskorn (2014), in his review published on the same issue (Alexander and Preskorn, 2014). They pointed out that even the first line antidepressants such as the selective serotonin reuptake inhibitors (SSRIs) and serotonin-norepinephrine reuptake inhibitors have limitation like poor response and remission rates, slow onset of action, poor tolerability, persistent adverse effects, and the potential for clinically significant pharmacokinetic drug interactions. It is, therefore, the novel antidepressant is required to be free from above limitations.

Alkaloids are a group of naturally occurring chemical compounds that contain mostly basic nitrogen atoms. This group also includes some related compounds with neutral (McNaught and Wilkinson, 1997) and even weakly acidic properties (Manske, 1965). Alkaloids are produced by a large variety of organisms, including bacteria, fungi, plants, and animals, and are part of the group of natural products (also called secondary metabolites). Many alkaloids can be purified from crude extracts by acid-base extraction. Many alkaloids are toxic to other organisms. They often have pharmacological effects and are used as medications, as recreational drugs, or in entheogenic rituals. The present review contained a detail note of the antidepressant activity of various plants alkaloids and are a candidate for further detail studies to evaluate their clinical utility.

## Alkaloids—as antidepressant agents

The antidepressant effect of various plant alkaloids has been reported in the literature (Table 1). Brazilian group of researcher isolated strictosidinic acid from *Psychotria myriantha* Mull. which exhibited antidepressant-like effect when studied on a 5-HT system in rat hippocampus (Farias et al., 2012). Lee et al. (2012) showed that berberine administration significantly decreased immobility and increased climbing behavior in the Forced swim test. However, there was no effect on swimming time, while increased open-arm exploration in the elevated plus maze test which confirmed that the antidepressant-like activity (Lee et al., 2012). Martínez-Vázqueza et al. (2012) isolated certain alkaloids from *Annona cherimolia*, including 1,2-dimethoxy-5,6,6a,7-tetrahydro-4H-dibenzoquinoline-3,8,9,10-tetraol, anonaine, liriodenine, and nornuciferine. The results showed that repeated treatment with this plant produced an antidepressant-like action in mice (Martínez-Vázqueza et al., 2012). The β-carboline alkaloids such harmane, norharmane, and harmine dose-dependently reduced the immobility time in the mouse forced swim test and thus produced an antidepressant-like effect (Farzin and Mansouri, 2006). A team of the researcher isolated Akuammidine, rhaziminine, and tetrahydrosecamine from *Rhazya stricta*. Acute administration of the lyophilized extract of *R. stricta* resulted in a significant antidepressant-like effect in experimental animals (Ali et al., 1998). Idayu and his co-researchers from Malaysia (2011) isolated mitagynine as an active component of *Mitagyna spicosa*. Mitagynine i.p injection significantly reduced the immobility time of mice in both forced swim test and tail suspension test without any significant effect on locomotor activity (Idayu et al., 2011). A team from the Republic of China outlined Mauritine A as an active compound found in *Ziziphus apetala* which showed strong activity against 11-β-hydroxysteroid dehydrogenase inhibition *in vitro* (Han et al., 2011). The diterpene alkaloids (Napelline, songorine, hypaconitine, and mesaconitine) has been isolated from *Aconitum baicalens* exhibited an antidepressant-like effect in an animal model of depression (Nesterova et al., 2011). An Dhingra and Valecha (2014) isolated Punaravine an alkaloid from *Boerhaavia diffusa* Linn. It showed significant antidepressant activity in unstressed and stressed mice in different models (Dhingra and Valecha, 2014). Jiang et al. (2015) purified Evodamine from *Evodia fructus* and found that it could reverse the decreases of sucrose preference, a number of crossing, 5-HT, and Na level and also increase immobility time (Jiang et al., 2015). A group of researcher led by Loria et al. (2014) from the USA established that Mesembrine present in *Sceletium tortuosum* have antidepressant property in animal studies (Loria et al., 2014). Wattanathorn and his field workers from Thailand assessed that Piperine, a major alkaloid isolated from *Piper nigrum*. Its antidepressant activity has been observed in mice exposed to both chronic and acute stress. It caused a significant change in both immobility and swimming times (Wattanathorn et al., 2008). A team from the China School of Pharmacy isolated Leatispicine, an amide alkaloid from *Piper laetispicum*. When tested in the forced swim test, it caused a significant dose-dependent decrease in mobility at various test doses and thus possessed antidepressant activity (Yao et al., 2009). Xu and coworkers obtained protopine from a Chinese plant, *Dactylicapnos scandens* Hutch, had an antidepressant effect in mice. It dose-dependently reduced the immobility time in the tail suspension test and thus could be effective for the moderate state of depression (Xu et al., 2006). Addition to all those, pramipexole is a non-ergoline alkaloid showed significant clinical efficacy in a double-blind, placebo-controlled study in bipolar and unipolar depressive patients (Zarate et al., 2004).

**Table 1:** **List of isolated alkaloids with plant alkaloids with antidepressant effect**.

**Plant name**	**Structure**	**Mechanism**	**References**
*Berberis aristata*	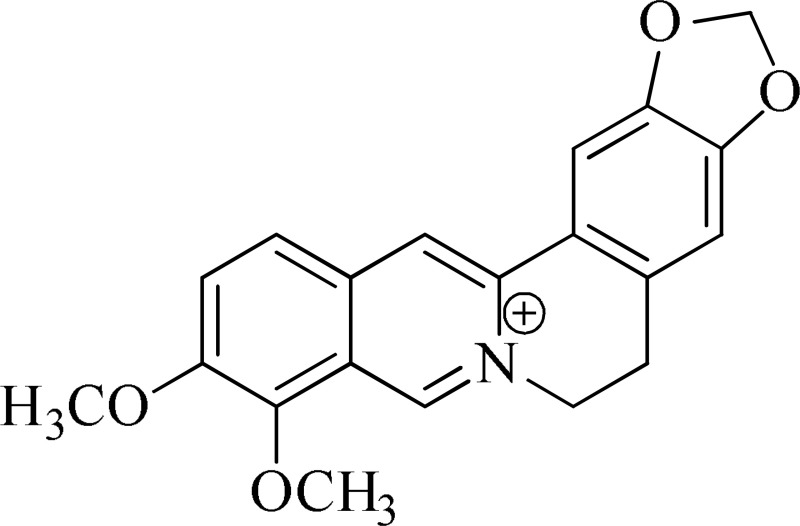 Berberine	Serotonergic, noradrenergic, and dopaminergic interventions	Kulkarni and Dhir, 2008; Lee et al., 2012
*Psychotria myriantha*	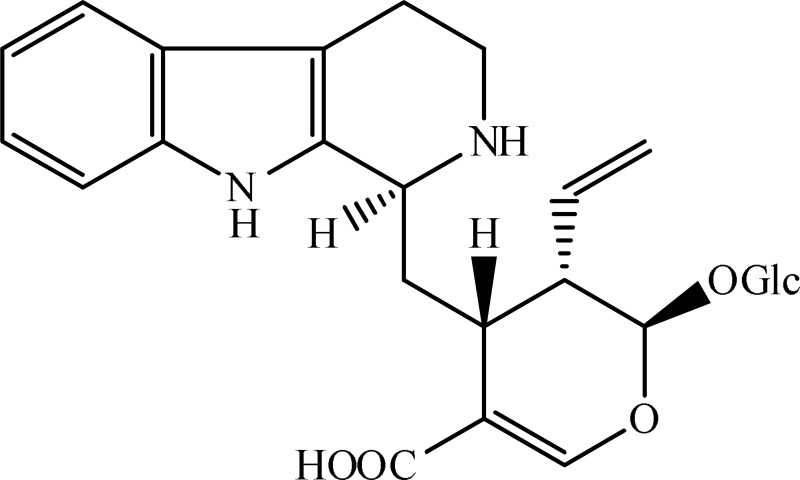 Strictosidine acid	MAO inhibition	Farias et al., 2012
*Annona cherimolia*	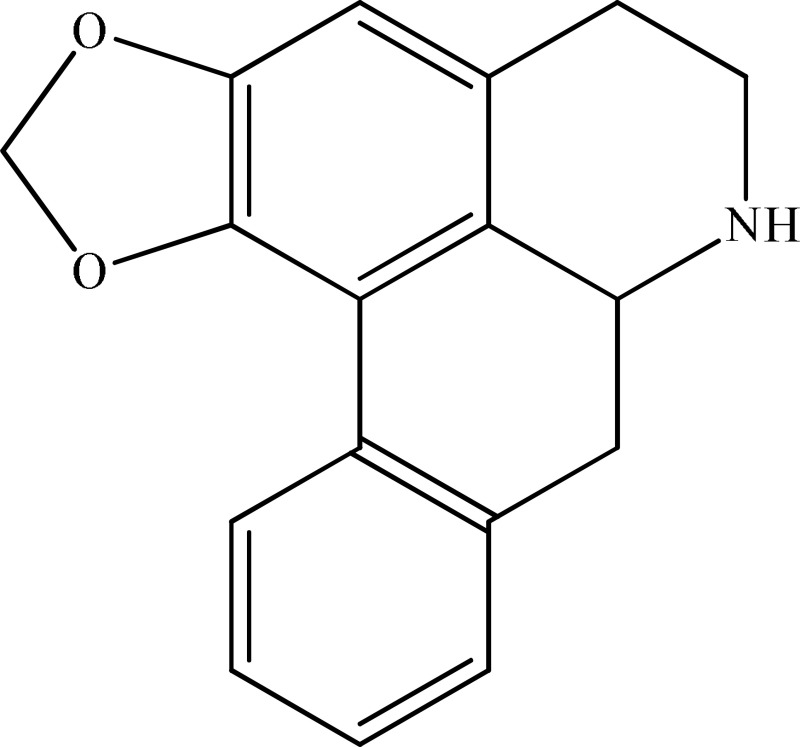 Anonaine 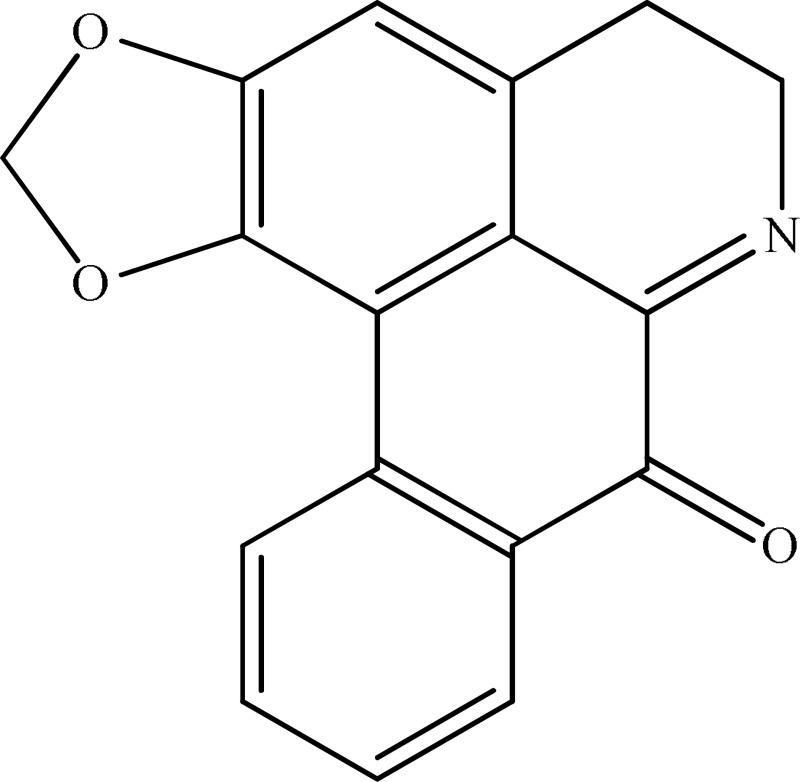 Liriodenine	Increase in monoaminergic turnover	Martínez-Vázqueza et al., 2012
*Rhazya stricta*	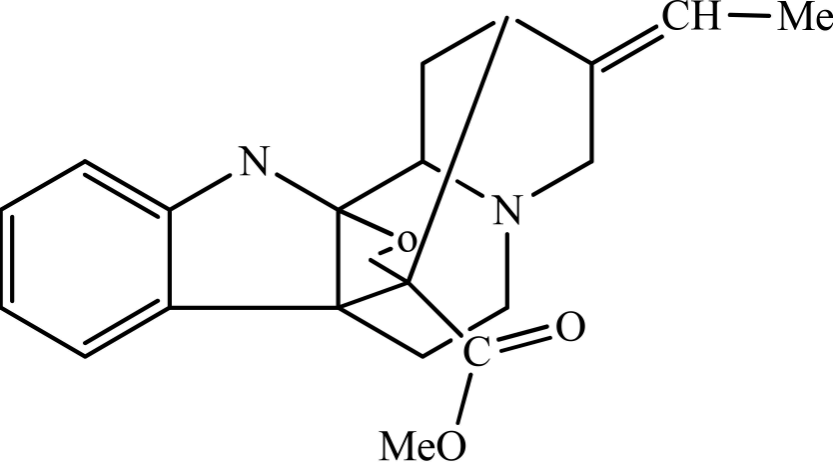 Akuammine	MAOA inhibition	Ali et al., 1998
*Mitagyna speciosa*	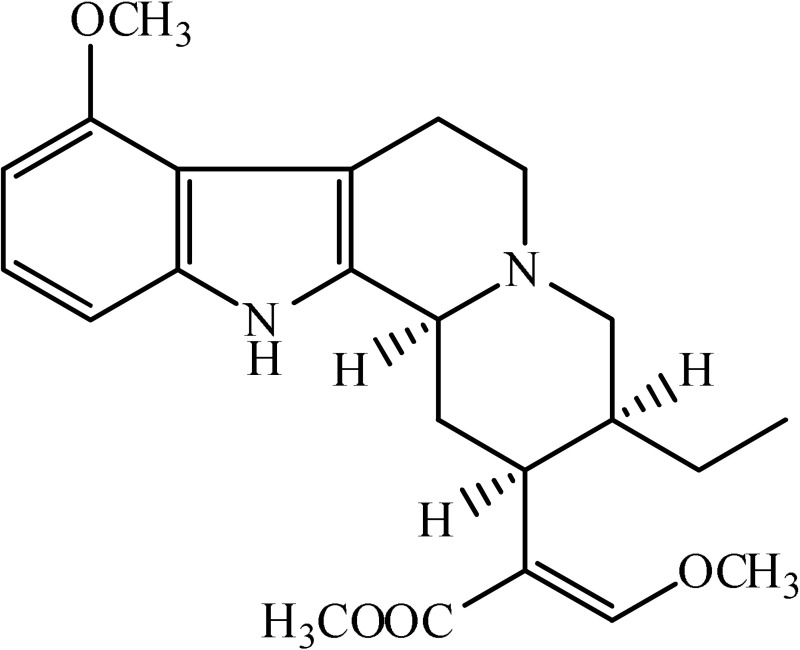 Mitagynine	Reducing the release of corticosterone	Idayu et al., 2011
*Peganum harmala*	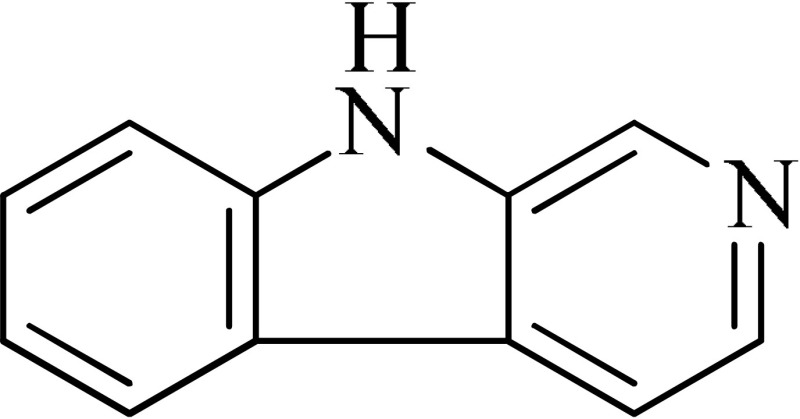 Norharmane 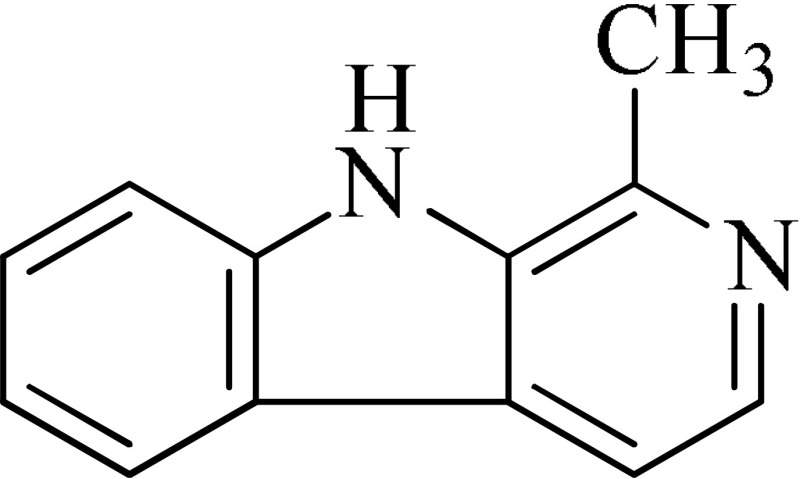 Harmane 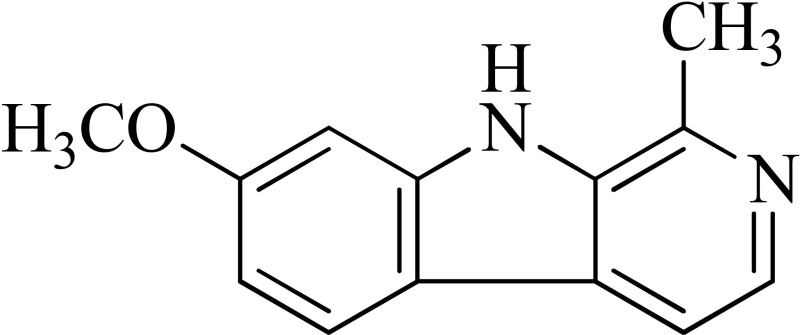 Harmine	Interfering with MAO-A and several cell-surface receptors, including serotonin receptor 2A	Farzin and Mansouri, 2006
*Ziziphus apetala*	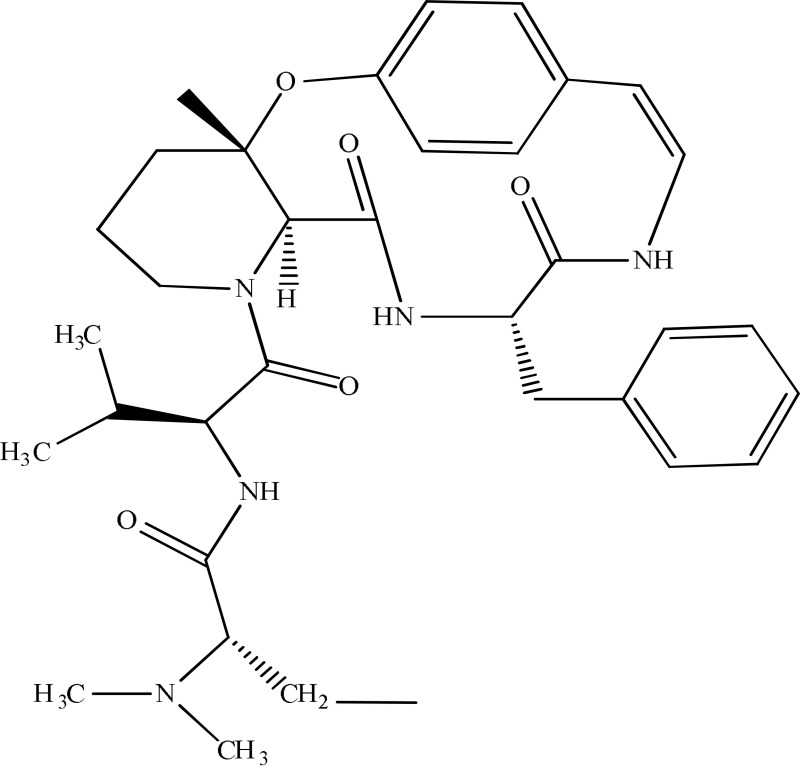 Mauritine A	11-β-hydroxysteroid dehydrogenase inhibition	Han et al., 2011
*Aconitum baicalense*	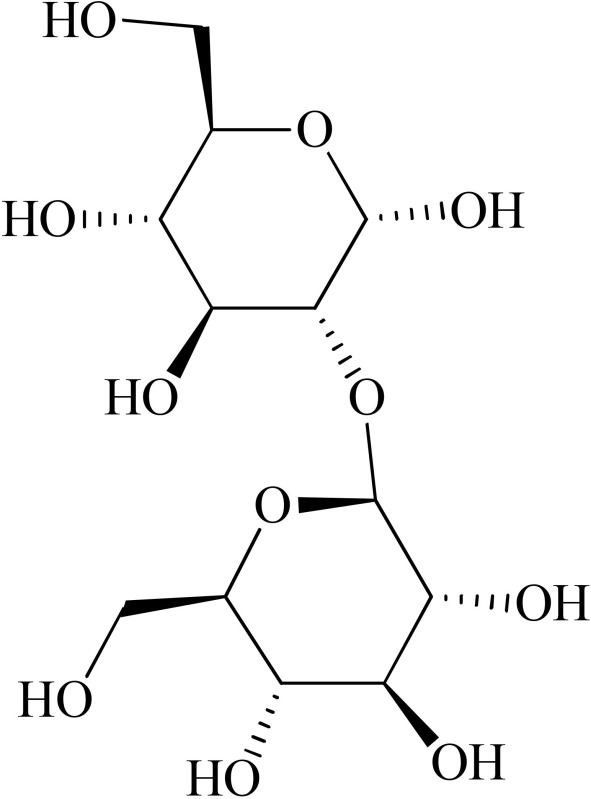 Songorine	Improved serotonergic system	Nesterova et al., 2011
*Boerhaavia diffusa*	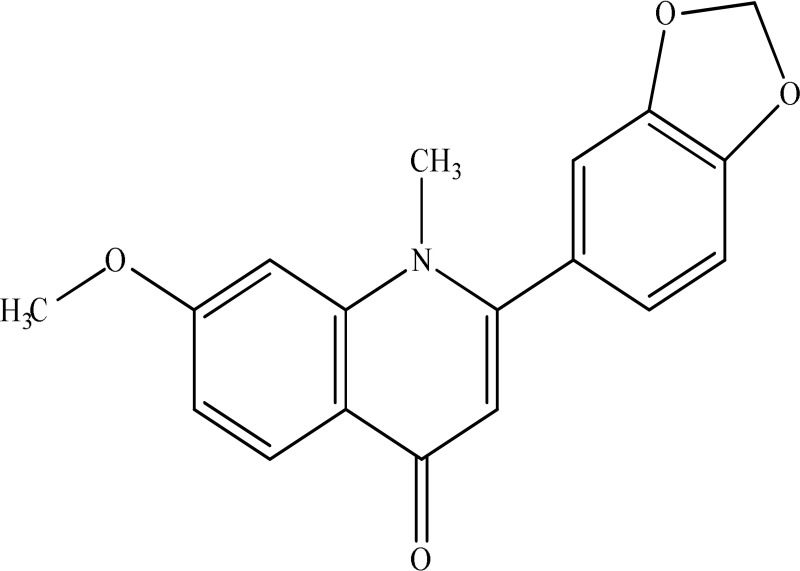 Punarnavine E	MAO inhibition and decreased plasma corticosterone level	Dhingra and Valecha, 2014
*Evodia fructus*	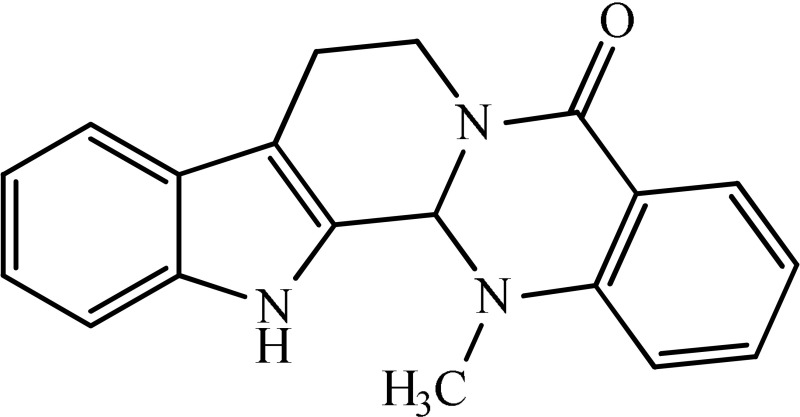 Evodiamine	Effects on the monoamine transmitters and BDNF-TrkB signaling in the hippocampus	Jiang et al., 2015
*Sceletrium tortuosum*	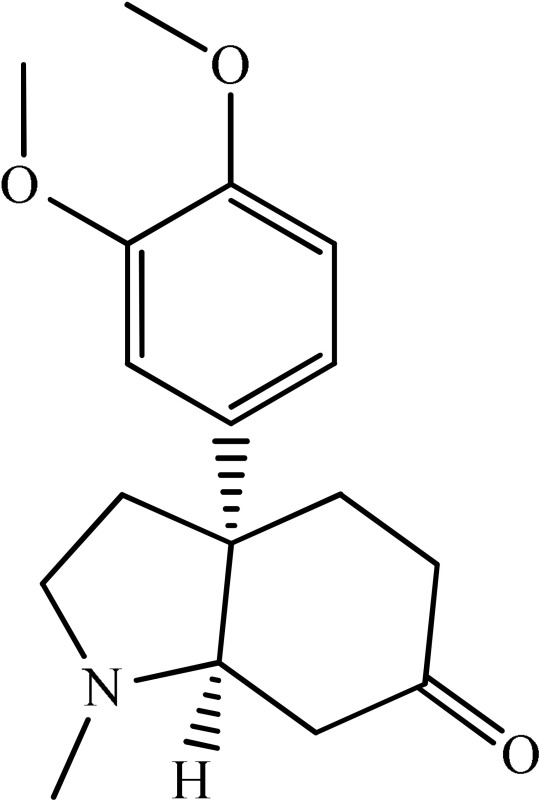 Mesembrine	5HT reuptake inhibition	Loria et al., 2014
*Piper nigrum*	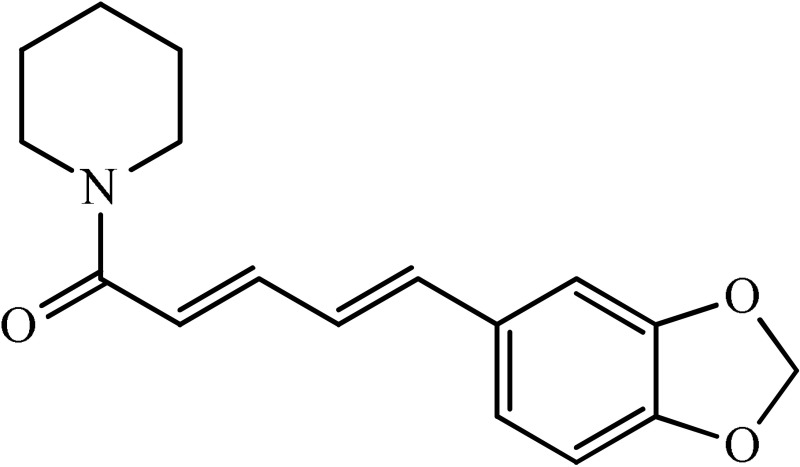 Piperine	Inhibition of MAO enzymes, elevation of brain 5-HT and BDNF levels	Wattanathorn et al., 2008
*Piper laetispicum*	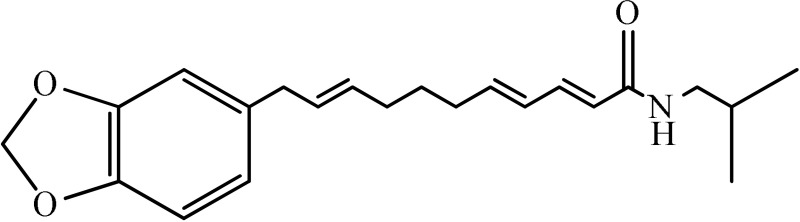 Leatispicine 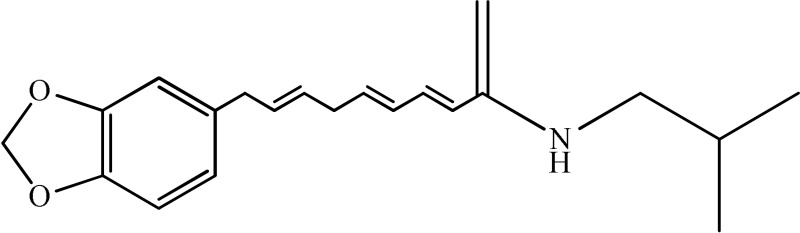 LeatispiamideA	Mechanism not studied yet	Yao et al., 2009
*Dactylicapnos scanens*	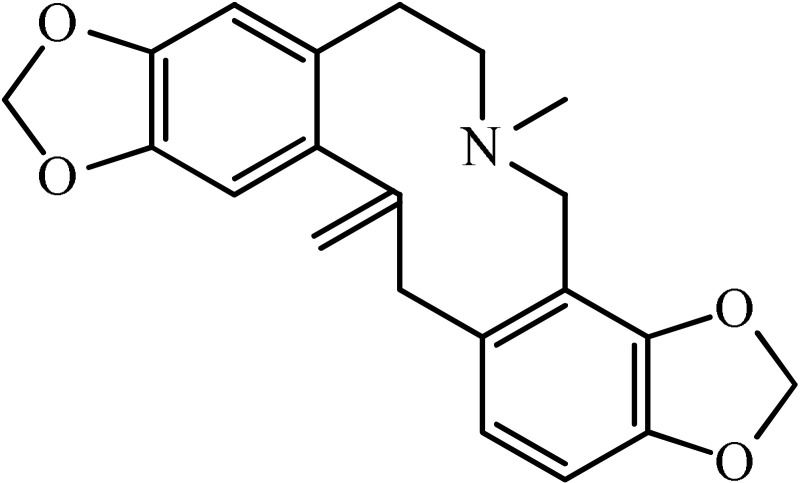 protopine	Inhibition of serotonin transporter and Noradrenaline transporter	Xu et al., 2006

## Antidepressant mechanism (s) of plants alkaloids

Strictosidinic acid probably exhibited antidepressant effect due to an inhibition of monoamine oxidase activity (Farias et al., 2012). 1,2-dimethoxy-5,6,6a,7-tetrahydro-4H-dibenzoquinoline-3,8,9,10-tetraol, anonaine, liriodenine, and nornuciferine produced an antidepressant-like action from a generalized increase in monoaminergic turnover (Martínez-Vázqueza et al., 2012). Berberine, an isoquinoline alkaloid of barberry demonstrated antidepressant like effect mediated through serotonergic, noradrenergic, and dopaminergic intervention (Kulkarni and Dhir, 2008; Lee et al., 2012). The β-carboline alkaloids (harmane, norharmane, and harmine) provoked antidepressant-like effects by interfering with MAO-A and several cell-surface receptors, including serotonin receptor 2A (Glennon et al., 2000; Kim et al., 2001). Akuammidine, rhaziminine, and tetrahydrosecamine possible mechanism resulted in a biphasic effect on the MAOA inhibitory component of tribulin (Ali et al., 1998). Mitagynine exhibited antidepressant-like action by reducing the release of corticosterone (Idayu et al., 2011). Mauritine A show strong activity against 11-β-hydroxysteroid dehydrogenase inhibition *in vitro* and, therefore, could be the possible mechanism for its antidepressant effect (Han et al., 2011). The diterpene alkaloids of *Aconitum baicalense*, improved serotonergic system activity in an animal model of depression (Nesterova et al., 2011). Punaravin E acts probably through inhibition of MAOA activity in the brain and, in addition, it also showed antidepressant activity possibly through decreased plasma corticosterone level (Dhingra and Valecha, 2014). The underlying mechanism of Evodamine can be potentially linked to their modulating effects on the monoamine transmitters and BDNF-TrkB signaling in the hippocampus (Jiang et al., 2015). Mesembrine an alkaloid elicited its antidepressant effect by inhibiting 5HT reuptake (Loria et al., 2014). Piperine is reported to increase the serotonin level in the cerebral cortex and limbic areas and thus produced the anti-depression like activity. However, further investigations about precise underlying mechanism are still required (Wattanathorn et al., 2008). Leatispicine mechanisms of action were presumed to be acting on the central nervous system monoaminergic neurotransmitters (Yao et al., 2009). Protopine is identified as an inhibitor of both serotonin transporter and noradrenaline transporter *in vitro* assays (Xu et al., 2006).

## Conclusion

Our review on the basis of available literature suggested that alkaloids could play a potential role as natural antidepressants. Keeping in mind their abundance in nature, the alkaloids could be an economical source of healing the depressive disorder. The available therapeutic agents fail to produced effect in all patients; approximately 30–40% failure has been reported to first-line antidepressant drugs accompanied by the very slow onset of action. Several alkaloids are in clinical practice and producing outstanding results in different therapeutic classes. These reported alkaloids though evoked antidepressant effects in various animal studies, but still deficient in clinical evidence. In conclusion, enough scientific evidence gathered in our review supported that the plant-based alkaloids can serve as leads for antidepressant drug discovery. It is key to subject these alkaloids to further clinical studies for efficacy, potency, and safety to ensure their clinical status.

## Author contributions

SP carried out all the literature survey and written an initial draft of the review and AK draw the structures of compounds and help in review-draft. HK supervised the overall project and finalized the final version of the review.

### Conflict of interest statement

The authors declare that the research was conducted in the absence of any commercial or financial relationships that could be construed as a potential conflict of interest.
